# Pulpal response to partial pulpotomy versus full pulpotomy procedures in puppies: An experimental study

**DOI:** 10.1371/journal.pone.0312076

**Published:** 2024-12-12

**Authors:** Thiyezen Abdullah AlDhelai, Zeyad Alsughier, Faraj Alotaiby, Mustafa Hussein Alattas, Muhammad Qasim Javed, Madeh Sadan, Rania M. Salem, Mona A. Elkateb

**Affiliations:** 1 Department of Orthodontic and Pediatric Dentistry, College of Dentistry, Qassim University, Buraydah, Saudi Arabia; 2 Department of Oral and Maxillofacial Diagnostic Sciences, College of Dentistry, Qassim University, Buraydah, Saudi Arabia; 3 Department of Conservative Dental Sciences and Endodontics, College of Dentistry, Qassim University, Buraydah, Saudi Arabia; 4 Department of Clinical Sciences, College of Veterinary Medicine, Qassim University, Buraidah, Saudi Arabia; 5 Boston University Henry M. Goldman School of Dental Medicine, Boston, Massachusetts, United States of America; 6 Department of Pediatric Dentistry and Dental Public Health, Faculty of Dentistry, Alexandria University, Alexandria, Egypt; 7 College of Dentistry, Princess Nourah Bint Abdulrahman University, Riyadh, Saudi Arabia; Universidade de Trás-os-Montes e Alto Douro: Universidade de Tras-os-Montes e Alto Douro, PORTUGAL

## Abstract

**Background:**

A Partial pulpotomy technique is an alternative choice of treatment for immature permanent teeth with exposed vital pulps. This study aimed to compare the histopathological pulpal response of the primary teeth of puppies after partial pulpotomy and full pulpotomy using mineral trioxide aggregate (MTA).

**Materials and methods:**

72 primary premolars from experimental puppies aged 6–8 weeks were equally divided into test and control groups, as MTA partial pulpotomy (MTA-PP) and MTA full pulpotomy (MTA-FP). After 60 days, the teeth were extracted and examined histologically. The specimens were assessing the extent and intensity of inflammatory response (acute or chronic), necrosis, fibrosis, calcific bridge (presence or absence), pulp calcification, and pathological root resorption.

**Results:**

The Inflammation intensity was lower (2.8%) in MTA-PP than in MTA-FP (16.7%) (P = 0.004). MTA-FP also had a significantly higher percentage of localized (27.8%) and diffuse (11.1%) necrosis (P = 0.014), whereas both groups (P = 0.290) had an equal fibrosis degree. A calcific bridge was observed in 72.2% of cases. Meanwhile, pathological internal root resorption was more evident in MTA-FP (63.9%) than in MTA-PP (19.4%) (P <0.0001).

**Conclusion:**

Partial MTA pulpotomy procedure demonstrated promising histological findings that can be applied to vital-pulp therapy in primary teeth.

## Introduction

Pulp preservation is a primary goal for the treatment of Primary dentition. Therefore, vital-pulp therapy is a critical procedure aimed at maintaining and preserving the vitality of primary tooth pulp until the time for its exfoliation. The American Academy of Pediatric Dentistry (AAPD) recommends three treatment modalities for vital-pulp therapy. These modalities are indirect pulp treatment, direct pulp capping, and pulpotomy [1].

For mechanical and/or carious pulp exposure in primary teeth having reversible pulpitis or normal pulp, the procedure of choice is pulpotomy [1]. Formocresol pulpotomies for primary teeth over 12 months show favorable outcomes according to radiographic and clinical studies [2], although several disadvantages have also been noted. For example, Coll et al. [3] conducted a systematic review and meta-analysis. They concluded that the success rate of most pulpotomy procedures not using mineral trioxide aggregate (MTA) decreases to 70% or less after three years. Another study has reported the early exfoliation of pulpotomized teeth [4]. Based on histopathological studies on formocresol pulpotomies, variable pulp-tissue responses reportedly include mild to moderate or severe inflammation, internal resorption, calcific metamorphosis, fibrosis, and necrosis [5, 6]. On the other hand, evidence-based research indicates that MTA is superior to the most commonly used pulp-capping materials; thus, MTA should be deemed as the gold-standard pulpotomy agent for primary molars [7]. MTA is biocompatible, induces dentin-bridge formation, and promotes pulp-tissue regeneration [8]. Several researchers have also expressed apprehension in using pulpotomy as a treatment modality for extensive lesions in primary teeth given its invasive nature [3, 8]. Indeed, the AAPD acknowledges the urgency of identifying new techniques for vital-pulp therapy in primary teeth by using biocompatible medications [1].

In 1978, Dr. Cvek introduced partial pulpotomy (PP) to preserve pulp vitality in complicated crown fractured permanent teeth owing to trauma [9]. The technique has been subsequently recommended for use in permanent teeth with carious exposed pulp and controlled bleeding [1]. Unlike in pulpotomy, the cell-rich coronal pulp tissue is preserved in PP, so the healing and repair ability rates are higher [10]. A 92% success rate for PP in cariously exposed permanent teeth after two months has been reported in a systematic review and meta-analysis [11]. Previous studies have demonstrated that PP is less invasive and more effective in bleeding control than pulpotomy. The chance of clot formation, which may interfere with pulp healing, is also lower in PP [12]. Bakhtiar et al. [13] explored the pulpal response of permanent teeth to different materials used for PP. They found that in 56% of MTA PP-treated teeth, complete dentin-bridge formation without inflammation occurred after eight weeks. However, few studies have focused on the use of PP in primary teeth despite its success in young permanent teeth. Nematollahi et al. [14] conducted a randomized controlled trial and compared the clinical and radiographic success rates of MTA PP with those of formocresol pulpotomy. No significant differences are found between both procedures throughout a 24-month period. Similarly, Abdelhafez et al. [15] reported comparable success rates between various pulpotomy methods in pediatric dentistry.

There is a dearth of studies discussing the efficacy of partial pulpotomy and full pulpotomy procedures using various materials, including mineral trioxide aggregate (MTA), on human primary teeth. However, these studies often focus on clinical and radiographic evaluations rather than detailed histological assessments [14, 15]. Though human studies are invaluable, they are sometimes limited by ethical concerns, variability in patient compliance, and difficulties in obtaining sufficient sample sizes for histological examination.

The present study aims to address these gaps by using an animal model that allows controlled experimental conditions and detailed histological analysis. Puppies’ primary premolars were utilized due to the similarity of their dental development to human primary teeth, including the process of eruption and exfoliation. It allows the researcher to closely monitor the histopathological pulpal responses to partial and full pulpotomy procedures using MTA as a pulp medicament in a controlled environment, thereby providing insights directly translatable to pediatric dentistry. The null hypothesis was that there would be no significant difference in the histopathological response of the pulp tissue of primary teeth to partial pulpotomy (PP) and full pulpotomy (FP) using mineral trioxide aggregate (MTA).

## Material and methods

### Ethical statement

The approval of the Research Ethics Institutional Review Board (IRB) of Qassim University, Saudi Arabia (21-05-05) was obtained for the current research protocol on 15 December 2021. Puppies were acquired, maintained, and treated according to the guidelines of the Laboratory Animal Control Guidelines of Qassim University based on the Guide for the Care and Use of Laboratory Animals of the National Institutes of Health (NIH). The disposal of all wastes and tissues used in the experiment followed the standard methods approved by the institution through the University Veterinary Hospital.

### Study design

The study design was an experimental animal split-mouth type. A total of 72 primary premolars (as defined in veterinary dentistry literature, which refers to the temporary premolars of the puppies) were obtained from six puppies. The MTA full pulpotomy (MTA-FP) was the control group, while the MTA partial pulpotomy (MTA-PP) was the test group. MTA-PP was applied to the premolars on one side of the arch, and MTA-FP was used on the opposite side.

### Sample size

The sample size was estimated based on assuming a 5% alpha error and 80% study power. According to Bakhtiar et al, [13], after 2 months, there was no signs of inflammation in 100% of teeth treated with MTA Partial Pulpotmy. While no inflammation was seen in 75% of teeth that had MTA Pulpotomy treatments [16]. Based on the difference between two independent proportions, the minimum sample size was calculated as 28 teeth per group, increased to 36 teeth to compensate for processing errors after comparing proportions using G power (Total sample = 72 samples).

### Experimental animals

This study included six male mixed-breed healthy puppies obtained from the Qassim Laboratory Animal Farm (Qassim, Saudi Arabia). They were aged 6–8 weeks and weighed 3–3.8 kg. They were free from cracks or dental caries as confirmed upon clinical examination by a veterinarian.

### Housing and husbandry

The puppies were maintained in hard-sided cages lined with soft blankets with the temperature controlled to 20°–25°C in the animal facility of the University Veterinary Hospital of Qassim University. The puppies were fed a balanced diet including bread and milk three times a day. Water and food were withheld 12 hours prior to the operative procedure.

### Allocating animals to experimental groups

An independent trial person randomly divided intact examined teeth according to The Modified Triadan System [17]. Then, they divided equally into two groups (36 teeth each) by using a random-number generator computer software (Random allocation software version 1.0.0, M. Saghaei, Isfahan University, Iran). MTA-FP was conducted on teeth on the right quadrant of the upper arch, whereas MTA-PP was performed on teeth on the left side. The opposite was implemented on the lower arch. Group allocation was written on paper, which was folded and enclosed in a sealed envelope identifying the puppy number. An assistant opened the envelope at the intervention time.

### Experimental procedures

Before the formal research began, a pilot study was conducted to calibrate all procedures and familiarize the operator with the anatomical variations between different primary premolars. The study involved performing full and partial pulpotomy procedures on the extracted primary premolars (506, 507, 508, 706, 707, and 708) collected from a 4-month-old puppy under general anesthesia.

Treatment was performed in a room that was temperature controlled at 25°C. A veterinarian administered general anesthesia intravenously administration as follows: 0.5 mg/kg body weight (BW) l-methadone (Methadyne, Jurox Pty Limited, Rutherford, Australia), 0.5 mg/kg BW diazepam (Ziapam, TVM UK, Oxfordshire, United Kingdom), 2 mg/kg BW ketamine (10%; Dopalen, Ceva Saúde Animal Ltda, Paulínia–SP, Brazil), and 0.2 mg/kg BW xylazine (Xylased Bioveta, a.s, Ivanovice na Hané, Czech Republic). Teeth disinfection was conducted by rinsing with 0.2% chlorhexidine (Hexidine, Icpa Health Products Ltd, India). Cavity preparations from the occlusal surface was performed by an expert operator using a size #330 sterile carbide bur (SS White Dental, Lakewood, NJ, USA). In the PP group, pulp was exposed at the most prominent cusp tip’s pulp horn according to puppies teeth anatomy using a size #2 sterile round diamond bur (SS White Dental, Lakewood, NJ, USA) under the conditions of a high-speed handpiece and water cooling. Pulp was removed to the full depth of a #2 round bur (~2 mm) followed gentle irrigation using normal saline. Bleeding control was performed using a sterile, moist cotton pellet for 1 min. Then, by using MTA Applicator, the exposure site was covered by MTA paste of 3:1 water-to-powder ratio (MTA-Angelus, Londrina, Paraná, Brazil) based on the manufacturer’s instructions. Cavity restoration was accomplished using conventional glass ionomer cement (GIC; Cavex Glass Ionomer Cement, The Netherlands).

In the full pulpotomy group, the pulp chamber was completely deroofed by joining the pulp horn using a size #4 sterile diamond round bur (SS White Dental, Lakewood, NJ, USA), and the procedure was visually and tactically verified to ensure full access to the pulp chamber. Removal of the coronal pulp tissue was carefully performed under high magnification. The amputation up to the canal orifices was performed using a sterile, sharp spoon excavator (SS White Dental, Lakewood, NJ, USA). After irrigating the pulp chamber, bleeding was controlled as in the PP procedure, and MTA paste was gently applied over the pulp stumps. The cavity was then sealed with GIC restoration. The bur was replaced after every five tooth preparations for both approaches. Two months postoperatively, the puppies were anesthetized, and after teeth extraction, the soft tissue was sutured. Pain was controlled on the first day by subcutaneous injection of 0.05 mg/kg meloxicam (Metacam, Boehringer Ingelheim International GmbH, Ingelheim am Rhein, Germany). On the second day, 0.1 mg/kg BW metacam oral suspension was administered for one week. Then, these puppies return to the Qassim Laboratory Animal Farm.

The extracted teeth were stored in 10% formalin until histopathological study. The specimens were decalcified for 5 days with formalin–nitric acid (10 mL of formalin, 80 mL of distilled water, and 10 mL of nitric acid). After transferring the specimens into a 5% sodium sulfate neutralizing solution for 12 h, they were dehydrated in an ascending-amount series of ethanol for 2 h each followed by clearance with xylene. They were embedded in paraffin wax and serially sectioned longitudinally into 5 μm–thickness. Finally, the specimens were stained with hematoxylin and eosin (H&E) and analyzed under a light microscope (BX51, Olympus Corporation, US).

### Experimental outcomes measure

Herein, the present study considered the study outcome as the pulp-tissue response to the treatment procedures. A trained oral and maxillofacial pathologist blinded to all procedures examined all samples by using the coding system. After evaluating the slides twice at six-day intervals, intra-examiner reliability was determined using the kappa coefficient (range = 0.88–0.96), representing a nearly perfect agreement. The pulp-tissue reaction to treatments was evaluated based on modified criteria of Jabbarifar et al.’s [16] modified criteria (Table 1).

**Table 1:** Criteria of assessment of pulpal response to capping materials.

***Criteria of assessment***		**Scores**			
		Score 0	Score 1	Score 2	Score 3
***Inflammation***	**Type of Inflammation**	No signs of inflammation	Acute: cells are mostly PMNs	Mixed: cells including PMNs (acute), lymphocytes, and plasma cells (chronic)	Chronic: cells are lymphocytes and plasma cells
	**Intensity of Inflammation**	Absent of inflammatory cells	Mild: few focal or diffuse inflammatory cells seen in high power	Moderate: inflammatory cells seen in medium power	Severe: numerous inflammatory cells seen in low power
	**Hyperemia**	Absent/Normal	Mild: dilated blood vessels	Moderate: dilated, branching, and/or extravasated RBC	Severe: enlarged and congested blood vessels and/or significant extravasated RBC
***Calcified bridge***		Absent	Present (evidence of calcification)		
***Necrosis***		Absent	Present		
			Localized	Diffuse	
***Fibrosis***		Absent	Present		
			Localized	Diffuse	
***Pulp calcification***		Absent	Present		
***Pathological Internal root resorption***		Absent	Present		

### Statistical analysis

Data are presented as frequencies and percentages. Considering the split-mouth design, categorical variables (pulp calcification, calcific bridge presence or absence, and pathological root resorption) were compared using McNemar’s test. Ordinal variables (hyperemia, necrosis, inflammation type and intensity, and fibrosis) were compared using the Wilcoxon signed-rank test. "A P value of 0.05 was set as the significance level, and all tests were two tailed. Data were analyzed using SPSS for Windows version 23 (IBM Corp., Armonk, NY, USA).

## Results

Vital-pulp treatment was performed on 72 deciduous premolars. A total of 36 MTA-PP and 36 MAT-FP samples were obtained. The pulp-tissue response of MTA-PP was compared with that of MTA-FP (Table 2). Chronic inflammation (score 3) was higher in MTA-FP (66.7%) than in MTA-PP (47.2%), whereas acute and mixed inflammation (scores 1 and 2, respectively) was higher in MTA-PP (13.9%) than in MTA-FP (33.3%). No significant difference existed between the groups (P = 0.085). Inflammation intensity was less in MTA-PP, with only 2.8% demonstrating severe inflammation (score 3) compared with the 16.7% in MTA-P. The difference was statistically significant (P = 0.004). No significant differences existed between the test and the control groups among all hyperemia types (mild, moderate, and severe). Calcific-bridge presence was higher in MTA-PP (72.2%), but the difference was not statistically significant. Localized and diffuse necrosis were significantly higher (27.8% and 11.1%, respectively; P = 0.014) in the control than in the test group. A nearly equal distribution of fibrosis (scores 1 and 2) was observed between groups (P = 0.290). MTA-PP had higher pulp calcification (33.3%) than MTA-FP (16.7%), but the difference was not statistically significant. MTA-FP had greater pathological internal-root resorption (63.9%) than MTA-PP (19.4%) (P <0.0001).

**Table 2:** Comparison of pulp tissue response between MTA partial pulpotomy versus pulpotomy procedures.

Criteria of assessment		Scores	Test (MTA-PP) (n = 36)	Control (MTA-FP) (n = 36)	(*P* value)
**Inflammation**	Type of Inflammation	Absent	2 (5.6%)	0 (0%)	(0.085)
		Acute	5 (13.9%)	3 (8.3%)	
		Mixed	12 (33.3%)	9 (25%)	
		Chronic	17 (47.2%)	24 (66.7%)	
	Intensity of Inflammation	Absent	7 (19.4%)	2 (5.6%)	(0.004*)
		Mild	18 (50%)	14 (38.9%)	
		Moderate	10 (27.8%)	14 (38.9%)	
		Severe	1 (2.8%)	6 (16.7%)	
	Hyperemia	Absent	9 (25%)	10 (27.8%)	(0.713)
		Mild	18 (50%)	16 (44.4%)	
		Moderate	4 (11.1%)	7 (19.4%)	
		Severe	5 (13.9%)	3 (8.3%)	
Calcified bridge		Absent	10 (27.8%)	17 (47.2%)	(0.189)
		Present	26 (72.2%)	19 (52.8%)	
Necrosis		Absent	30 (83.3%)	22 (61.1%)	(0.014*)
		Localized	6 (16.7%)	10 (27.8%)	
		Diffuse	0 (0%)	4 (11.1%)	
Fibrosis		Absent	10 (27.8%)	8 (22.2%)	(0.290)
		Localized	24 (66.7%)	23 (63.9%)	
		Diffuse	2 (5.6%)	5 (13.9%)	
Pulp Calcification		Absent	24 (66.7%)	30 (83.3%)	(0.146)
		Present	12 (33.3%)	6 (16.7%)	
Pathological root resorption		Absent	29 (80.6%)	13 (36.1%)	(<0.0001*)
		Present	7 (19.4%)	23 (63.9%)	

* Statistically significant at P value <0.05

**Fig 1:**
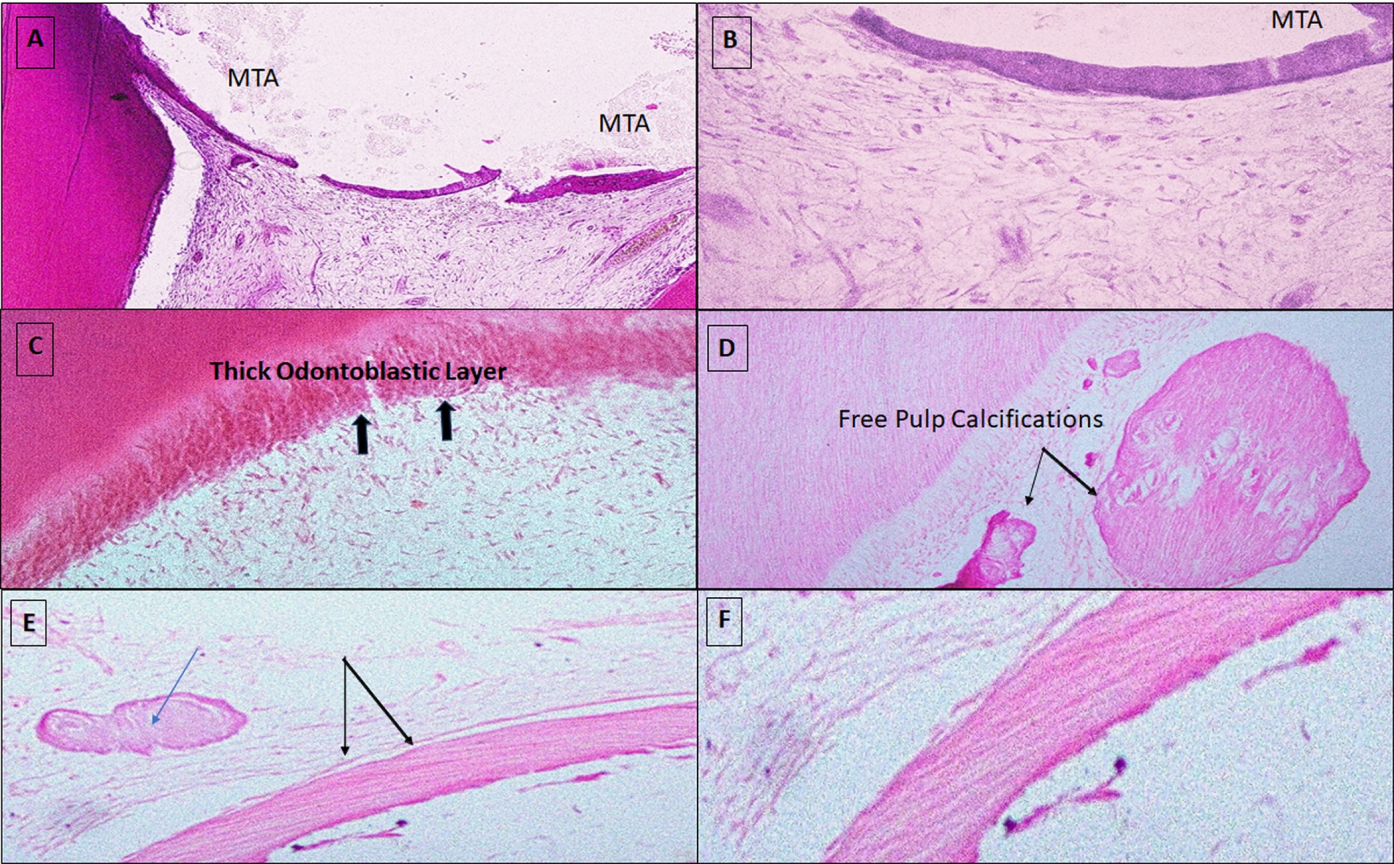
Pulp tissue response to MTA-PP (test group) after two months, showing **Fig 1A & 1B:** MTA material in direct contact with normal pulp tissue (H&E Fig 1. A x10 and 1. B X40). **Fig 1C:** Note the apparent normal thick odontoblastic layer (black arrows) (H&E × 40). **Fig 1D:** Evidence of normal dentinal tubules with multiple free pulp calcifications (H&E X20). **Fig 1E:** A noticeable regenerative calcified bridge (black arrows) adjacent to pulp calcifications (blue arrows) (H&E X10). **Fig 1F:** High power of the marked calcified bridge formation (H&E X40).

**Fig 2:**
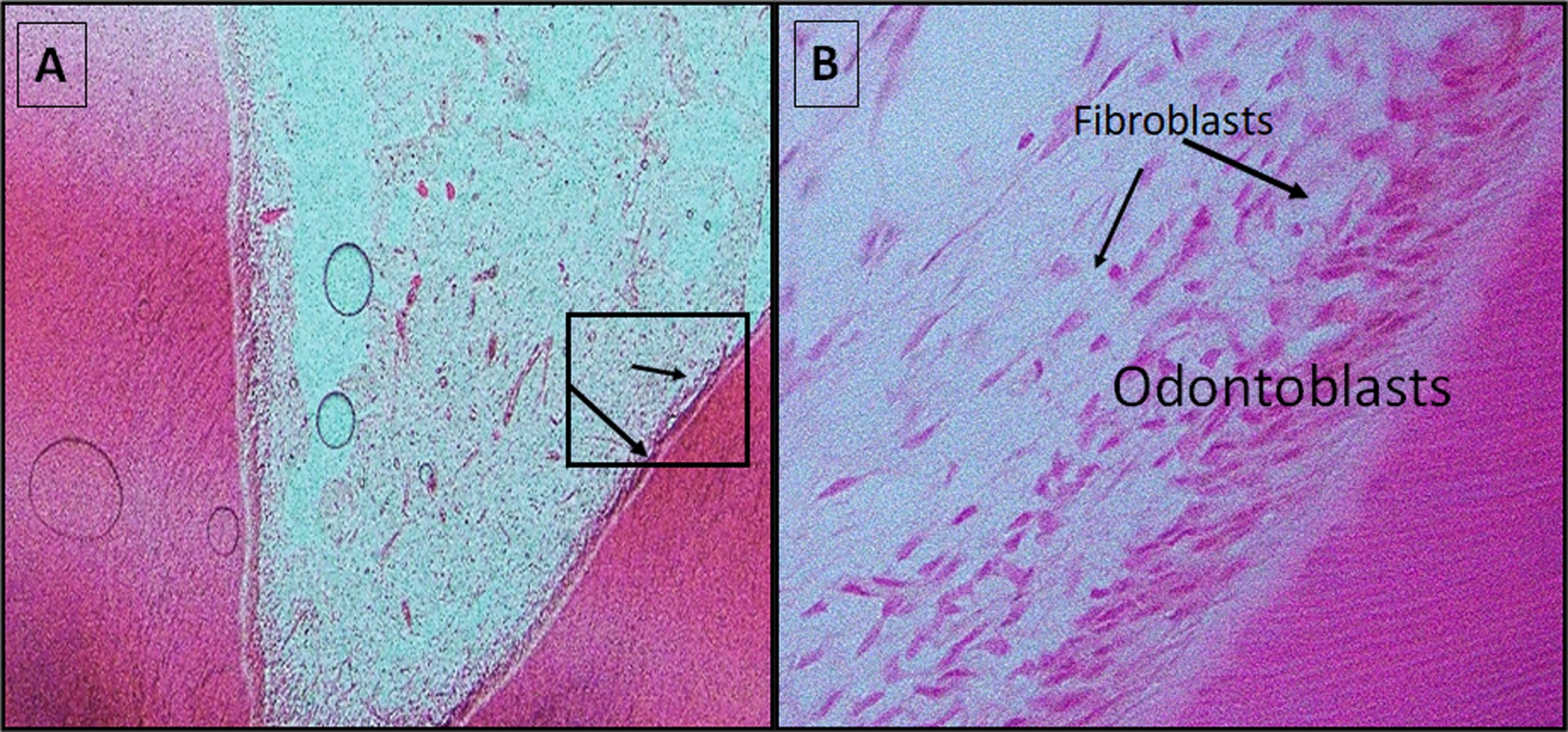
Pulp tissue response to MTA-FP (control group) after two months showing **Fig 2A:** apparently normal pulpal tissue with continuous odontoblastic layer (arrows) (H&E X10). **Fig 2B:** Note the preserved thick odontoblastic layer and abundant cell population of fibroblasts (arrows) (H&E X40).

Favorable pulp-tissue response was found 2 months after MTA-PP and MTA-FP procedures, as revealed by histopathological evaluation (Figs 1 and 2). Normal dentinal tubules appeared all over the dentin thickness. The odontoblast layer was preserved, and pulp was normal with no an apparent inflammation. Calcified-bridge presence was noted in MTA-PP (Fig 1E and 1F).

**Fig 3:**
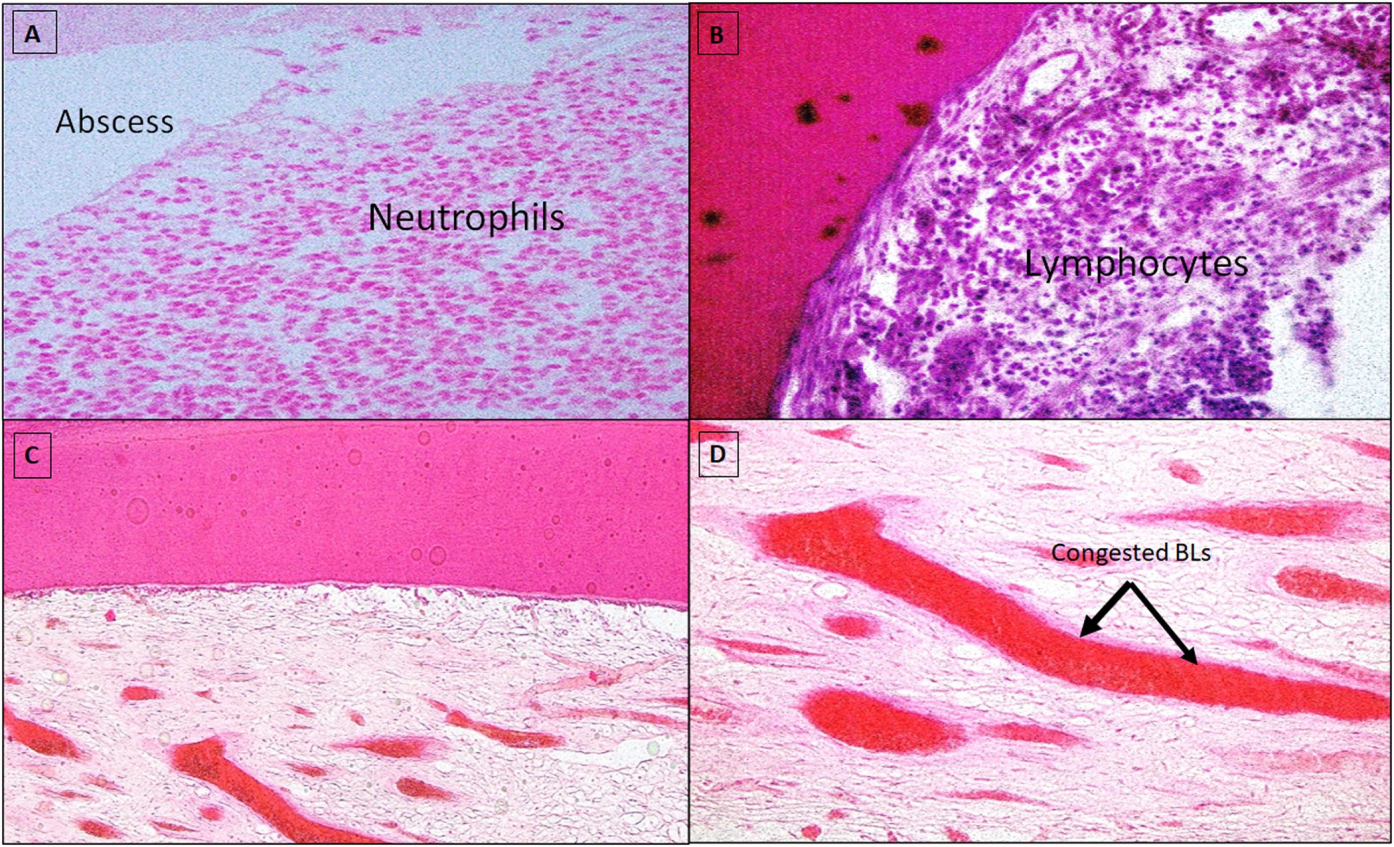
Unfavorable pulp tissue response two months after treatment. **Fig 3A:** MTA-FP (control group) presented a severe infiltration of acute inflammatory cells in the pulp tissue with neutrophil predominance and abscess formation (H&E X40). **Fig 3B:** Showed moderate collection of chronic inflammatory cells in the MTA-PP (test group) (H&E X40). **Fig 3C & 3D:** Note signs of hyperemia with congested blood vessels (black arrows) in the control group (H&E Fig 3C X20, and Fig 3D X40).

**Fig 4:**
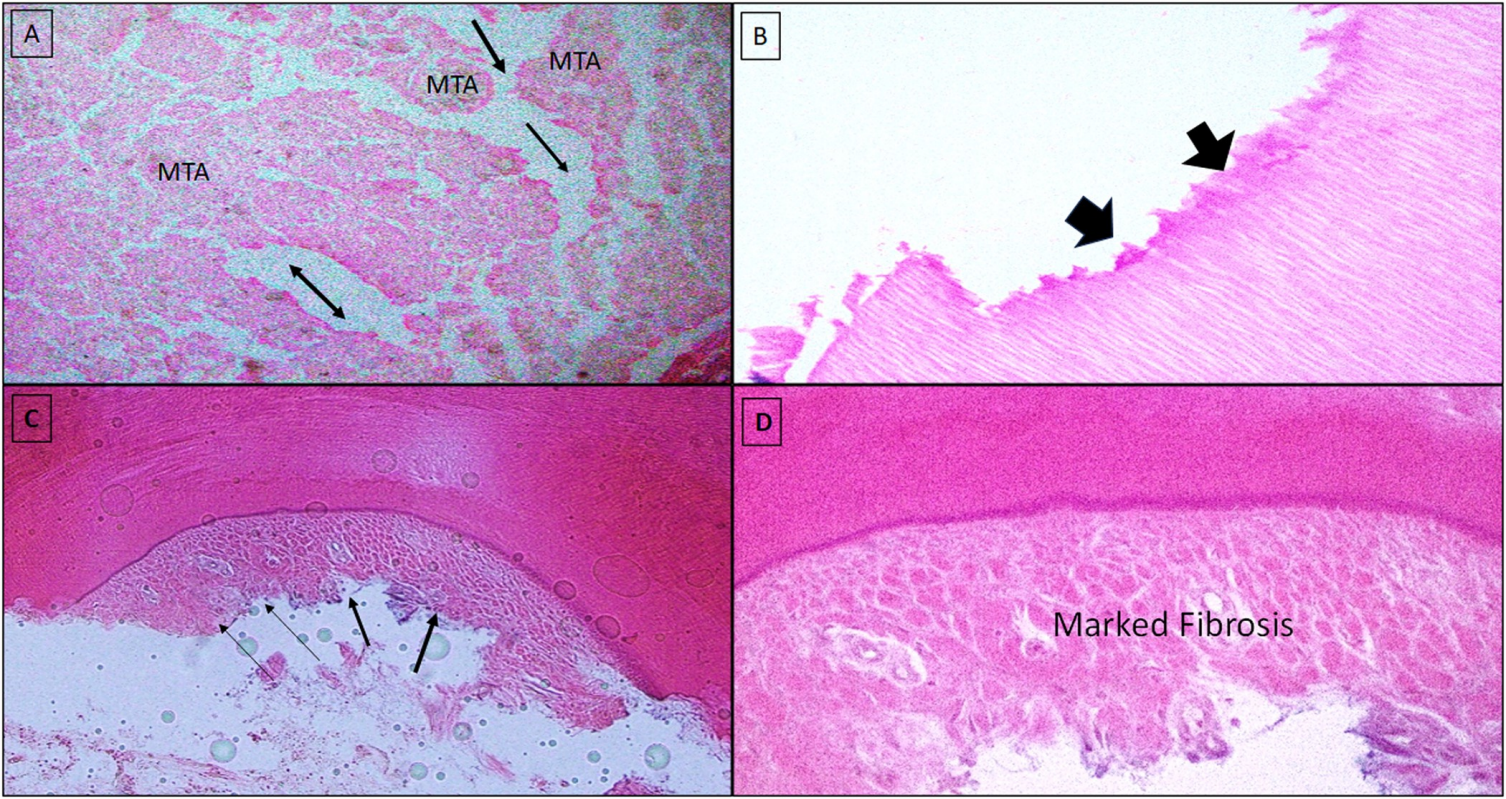
Unfavorable pulp tissue response to the MTA-FP (control group) showing **Fig 4A**. Signs of diffuse tissue necrosis (black arrows) are associated with the focal distribution of MTA material within the pulp tissue (H& E X10). **Fig 4B:** Note the pathological internal root resorption (black arrows) (H&E X40). **Fig 4C & 4D:** Showed marked fibrosis (black arrows) along the pulpal side of the dentinal wall (H&E Fig 4C X10, and Fig 4D X40).

Meanwhile, different inflammation degrees (i.e., mild to severe) were observed in specimens with unfavorable pulp-tissue response. Both groups demonstrated blood-vessel congestion and dilatation, and the infiltration of acute and/or chronic inflammatory cells and abscess formation was significant (Fig 3). Remarkable fibrosis, pathological root resorption, and necrosis were also detected in a few pulpotomy-treated teeth (Fig 4).

## Discussion

PP is considered more conservative than full pulpotomy. [10] PP aims to preserve any remaining vital pulp tissue that is functioning and infection free [1]. A healing environment is likely to emerge once this goal is achieved. The development of mineralized tissues in the exposed area is possible and may trigger a reparative response.

The success rate of vital-pulp therapy in primary teeth is usually determined through radiographic and clinical evaluation. Nevertheless, the gold standard in determining such success is still histopathological examination [18]. To our best knowledge, the present work is the first experimental histologically based study that compared the influence of two MTA vital-pulp therapy techniques (pulpotomy and PP) on the primary tooth pulp of puppies. Given the difference in pulp-tissue responses between both procedures, the null hypothesis was hereby rejected.

The age of the puppies in this study was 6–8 weeks because this age range marks the completion of primary dentition. Considering that the shedding process of puppies’ primary teeth occurs beyond the fourth month of their life, the follow-up period was deemed to be only two months [19]. MTA, a regenerative material with a high application-success rate, is considered to be the gold standard for vital-pulp treatment of primary teeth [20]. The design of the present experimental study was split mouth, i.e., both intervention techniques were applied to comparable sites to eliminate inter-subject variability. Standardized procedures were used in teeth treated in this work.

The pulpal responses to different techniques and medicaments have been extensively researched in experimental animals, but the main focus is on permanent teeth [21–24]. Studies on human teeth have also been performed for the same purpose, and pulpotomy is the most widely evaluated strategy [25]. Accordingly, the results on PP were compared with those obtained from permanent teeth."

After 2 months of follow-up, chronic inflammation (score 3) was noted in MTA-P-treated primary teeth and, to a lesser extent, in MTA-PP-treated ones. Additionally, the pulpotomy group had significantly higher inflammation intensity. The observed inflammation may be associated with the surgical trauma and the irritating effect of the capping material [26], which may also explain the increased inflammation intensity correlated with the higher invasiveness of pulpotomy. The study findings coincided with those of Lee et al. [22], Kang et al. [23], and Agamy et al. [25]. Lee et al. [22] evaluated PP on permanent teeth in experimental animals, whereas Kang et al. [23] experimentally studied pulpotomy techniques for permanent teeth. Notably, inflammation intensity was significantly higher in MTA-P. The success of MTA vital-pulp therapy is known to depend on the presence or absence, as well as the intensity and type, of the inflammatory response and the formation of a calcific barrier [27]. Therefore, the pulp response can be considered to be more favorable in primary teeth treated with partial than full pulpotomies.

Regarding hyperemia, all scores (mild, moderate, and severe) were almost the same, and no significant differences existed between teeth treated with partial and full pulpotomies. Mild hyperemia (score 1) achieved the highest score, confirming earlier findings about pulp-tissue response to MTA pulpotomies [22–24].

Calcific-bridge presence was higher (72.2%) in MTA-PP than in MTA-FP (52.8%). Although the difference was not statistically significant, it can be considered a sign of success for MTA-PP. Inflammatory cells reportedly play a crucial role in tissue healing and repair. The healing process is supported by macrophages producing various growth factors and cytokines. Proper balance must exist between pro- and anti-inflammatory cytokines for healing promotion and tissue repair post-injury [28]. Decreased infection or inflammation in the pulp enables regenerated odontoblast-like cells to differentiate and create reparative dentin or calcific bridges [29]. This finding was consistent with that of Bakhtiar et al. [13]. They observed complete dentin bridges in 56% of cases treated with MTA PP on permanent teeth scheduled for extractions. The study observation also agreed with that of Khalil et al. [21], but their study involves experimental rats and direct pulp capping.

In the current study, The test group demonstrated significantly less pulp-tissue necrosis than the control group, indicating that the response to treatment was more favorable. When at least one area of coronal pulp tissue demonstrates coagulation or liquefaction necrosis in any treated teeth, it is deemed as a primary indicator of pulp change that is irreversible [30]. The necrosis incidence is higher in the pulpotomy group, which may be due to the increased inflammation intensity. Balance must be maintained between inflammatory cells, which is crucial for healing. A disrupted inflammatory reaction can decrease the healing potential, resulting in a degeneration of the odontoblastic layer and even in the necrosis of pulp tissue. The necrosis (diffused type) score of 2 in MTA-FP was consistent with that found by Jabbarifar et al. [16], who found that necrosis was an unfavorable outcome of the procedure.

PP- and pulpotomy-treated teeth had a nearly identical fibrosis degree. Interestingly, fibrosis was absent in the experimental rats’ permanent-tooth pulp after MTA pulpotomy conducted by Salako et al. [31]. Their findings are the opposite of those observed in this current study, as 26 out of 28 fibrosis cases had scores of 1 and 2. The discrepancy may be explained by the different dentition types and experimental animals used. Rowe [32] showed a significant healing potential for rat pulp.

Although the test group had a higher pulp-calcification percentage than the control (33.3% and 16.7%, respectively), the coronal pulp had free/adherent pulp stones. This presence is considered a deviation from the normal histological features of healed pulp tissue [30]. The percentage observed was much lower than that reported by Jabbarifar et al. [16], in which the calcification rate in permanent teeth was 51.9% three months after MTA pulpotomy. The longer evaluation periods and different dentition types may explain the variation between the findings.

Pathological internal-root resorption was more prevalent in MTA-FP than in MTA-PP, further confirming MTA-PP superiority. Since these teeth were extracted before their physiological resorption time, the applied PP/FP technique might induce a histological response like internal resorption. The likelihood of internal resorption can be increased by certain factors such as reduced radicular pulp tissue, which decreases healing potential. The invasiveness of the procedure is another factor. Moreover, the vascularity of the apical region increases with physiologic resorption, which can attract odontoclasts and thus initiate internal resorption [26].

This study has some limitations. First is the inconsistent direction of sectioned teeth throughout the study. Second, is the absence of caries or inflammation in puppies’ teeth. Third, the decalcification of sectioned samples increases the difficulty and duration of the examination compared with conventionally H&E-stained histological sections. However, these findings can serve as a baseline for future experimental and clinical works. Accordingly, it is suggested that further clinical studies be conducted on human primary teeth to evaluate PP’s effectiveness and determine the suitable biological materials.

## Conclusion

Partial MTA pulpotomy procedure showed promising histological results that could be applied to vital pulp therapy in primary teeth.

## Supporting information

S1 File The ARRIVE guidelines checklist. (PDF)
